# Cross-Sectional Variations in Structure and Function of Coral Reef Microbiome With Local Anthropogenic Impacts on the Kenyan Coast of the Indian Ocean

**DOI:** 10.3389/fmicb.2021.673128

**Published:** 2021-06-23

**Authors:** Sammy Wambua, Hadrien Gourlé, Etienne P. de Villiers, Oskar Karlsson-Lindsjö, Nina Wambiji, Angus Macdonald, Erik Bongcam-Rudloff, Santie de Villiers

**Affiliations:** ^1^Pwani University Bioscience Research Centre (PUBReC), Pwani University, Kilifi, Kenya; ^2^Department of Biological Sciences, Pwani University, Kilifi, Kenya; ^3^Department of Animal Breeding and Genetics, Swedish University of Agricultural Sciences, Uppsala, Sweden; ^4^KEMRI-Wellcome Trust Research Programme, Kilifi, Kenya; ^5^Nuffield Department of Medicine, University of Oxford, Oxford, United Kingdom; ^6^Kenya Marine and Fisheries Research Institute, Mombasa, Kenya; ^7^School of Life Sciences, University of KwaZulu-Natal, Durban, South Africa; ^8^Department of Biochemistry and Biotechnology, Pwani University, Kilifi, Kenya

**Keywords:** coral reef microbiota, microbiome, COGs, human impacts, environmental stressors

## Abstract

Coral reefs face an increased number of environmental threats from anthropomorphic climate change and pollution from agriculture, industries and sewage. Because environmental changes lead to their compositional and functional shifts, coral reef microbial communities can serve as indicators of ecosystem impacts through development of rapid and inexpensive molecular monitoring tools. Little is known about coral reef microbial communities of the Western Indian Ocean (WIO). We compared taxonomic and functional diversity of microbial communities inhabiting near-coral seawater and sediments from Kenyan reefs exposed to varying impacts of human activities. Over 19,000 species (bacterial, viral and archaeal combined) and 4,500 clusters of orthologous groups of proteins (COGs) were annotated. The coral reefs showed variations in the relative abundances of ecologically significant taxa, especially copiotrophic bacteria and coliphages, corresponding to the magnitude of the neighboring human impacts in the respective sites. Furthermore, the near-coral seawater and sediment metagenomes had an overrepresentation of COGs for functions related to adaptation to diverse environments. Malindi and Mombasa marine parks, the coral reef sites closest to densely populated settlements were significantly enriched with genes for functions suggestive of mitigation of environment perturbations including the capacity to reduce intracellular levels of environmental contaminants and repair of DNA damage. Our study is the first metagenomic assessment of WIO coral reef microbial diversity which provides a much-needed baseline for the region, and points to a potential area for future research toward establishing indicators of environmental perturbations.

## Introduction

Coastal ecosystems are some of the most dynamic and vulnerable environments under various pressures from anthropogenic activities and climate change. Coral reef ecosystems, particularly, are of interest because of their importance for biodiversity, their productivity, and their worrisome decline globally.

The Western Indian Ocean (WIO) is an oceanic region in the warmest ocean (Indian Ocean) that is currently under pressure from global warming and increasing anthropogenic stressors, with unpredictable consequences, yet it is the least studied of all global oceans with respect to the associated microbial ecology (Díez et al., 2016). Indeed, the *Web of Science* records only four papers, published in the last decade (2010 – 2020) on genomic assessment of coral reef microbes in the WIO, compared to 17 in the Caribbean Sea and 114 in the Great Barrier Reef (GBR). Because of the broad variety of biogenic habitats and oceanographic conditions, the region’s waters encompass a diverse range of ecologically and nutritionally rich ecosystems (Van Der Elst et al., 2005). With its goods and services supporting over a quarter (more than 60 million people) of the region’s population who live within 100 km of the shoreline (Van Der Elst et al., 2005; Obura, 2017), the WIO and its coastal ecosystems are essential to the region’s economy. It is feared that with the rapidly increasing development and utilization of coastal zones (Neumann et al., 2015), the WIO’s resources are overwhelmed, overexploited and poorly managed, putting its wealth at risk. Indications of distress on the region’s ecosystems are noted with increasing frequency characterized by reduced fish catches, diminishing mangrove coverage, and declining coral reef cover (Van Der Elst et al., 2005; Obura, 2017) consequently threatening the livelihoods of dependent coastal communities. Protected areas are the principal tool of marine management in the region – the WIO countries have protected about 2.4 per cent of their marine area (Obura, 2017). Other strategies extensively applied by government institutions, Non-Governmental Organizations (NGO) and communities throughout the WIO to conservation coral reefs are reviewed by Hattam et al. (2020) and include the introduction of alternative livelihoods, coral gardening and payment for ecosystem services (PES) schemes. Monitoring of coral reef status in the WIO is undertaken under the Global Coral Reef Monitoring Network (GCRMN) umbrella, and has mainly involved visual estimations of hard coral cover, fish abundance and coral bleaching (Obura et al., 2019). Technological advancements including autonomous vehicles facilitating underwater, surface, and aerial surveys, and satellites, as well as structure from motion image processing, and acoustic techniques, are set to greatly improve monitoring of coral reef status. Advancements and declining costs of high-throughput sequencing technologies aided by autonomous reef monitoring systems has also improved collection of diversity data on cryptic marine communities for coral reef monitoring (Ransome et al., 2017). Marine microbes are thought to have significant potential as a cryptic community due to their rapid response to environmental change (Glasl et al., 2017).

Shifts in the composition and diversity of microbial communities may be a good indicator for a marine ecosystem’s health and for predicting stress responses (Won et al., 2017). Current efforts exploring the potential of microbes as indicators for coral reef ecosystem stressors often correlate taxonomic profiles with biogeographic variation (Glasl et al., 2017, 2018, 2019; Roitman et al., 2018). However, functional profiling may also be informative of environmental disturbance because similar community metabolism may comprise members of phylogenetically variable groups while communities comprised of similar taxa may differ in metabolic capabilities. Indeed, functional diversity has repeatedly been observed to predict ecosystem processes and properties better than taxonomic or phylogenetic diversity (Johnson and Pomati, 2020). Metagenomics provides the opportunity to access microbial community taxonomic and genomic content, as well as their functional potential, which can serve as indicators of the health in an ecosystem (Bellwood et al., 2019). For instance, comparative metagenomics studies have demonstrated that microbial diversity is influenced by the local environment (Behzad et al., 2016) leading to the hypothesis that unique environments harbor unique microbial diversity as well as unique metabolic pathways. Furthermore, Kelly et al. (2014) has shown that, at the ecosystem level, benthos determine core microbial taxa which then adapt to local oceanographic conditions by selecting for advantageous metabolic genes.

Coral reef microbial communities inhabit sediments, overlying water column, and benthic invertebrates such as corals and sponges (Bourne and Webster, 2013). Benthic-pelagic coupling occurs in shallow well-mixed tropical coral reefs where bacterial communities in benthic organisms, the sediments, and the overlaying water column are strongly interlinked (Bourne and Webster, 2013; Vanwonterghem and Webster, 2020). These interactions within the coral ecosphere are known to influence the composition of coral-associated microorganisms (Weber et al., 2019) through the benthos releasing host-specific microbes into the surrounding water, or producing dissolved organic matter which stimulates the activity of specific microbes within the surrounding water layer (Barott and Rohwer, 2012; Silveira et al., 2017; Walsh et al., 2017). Increases in the abundance of microbes in the reef water column has been correlated with an increase in coral disease and reduction in coral cover (Dinsdale et al., 2008; Bruce et al., 2012). Furthermore, since anthropogenic activities most likely impact coral health through the agency of the immediate pelagic and benthic surrounding, the water overlying corals and sediments are often the niches sampled to detect relevant signal in coral ecosphere (McDole et al., 2012; Bourne and Webster, 2013; Kelly et al., 2014; Tout et al., 2014; Walsh et al., 2017; Glasl et al., 2019). To approximate the anthropogenic impact on composition and function of coral reef microbiota, we compared cross-sectional metagenomes of near-coral seawater and sediment samples from three reefs subject to varied suites of environmental perturbations on the Kenyan coast of the WIO. This is the first shotgun metagenomic assessment of coral ecosphere on the WIO – previous studies explored the open water microbiome (Sunagawa et al., 2015) and virome (Williamson et al., 2012).

## Materials and Methods

### Study Sites and Human Activities

Site selection was guided by the Kenya Marine and Fisheries Research Institute (KMFRI) ensuring inclusion of locations in proximity to the range of human activities typical of cosmopolitan urban and rural settlements along the coast of Kenyan. These sites are spread across three of the five counties on the Kenyan coast.

**FIGURE 1:**
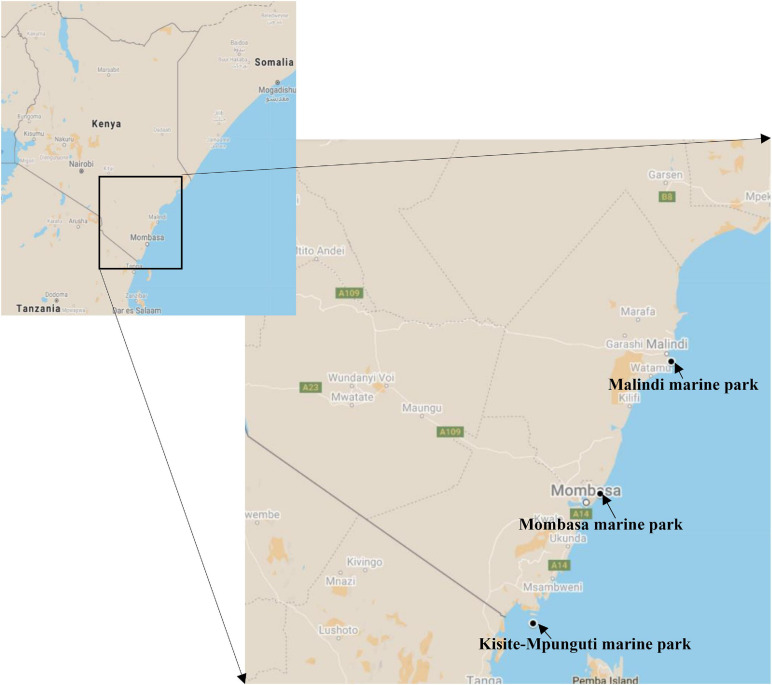
Map showing the location of the coral reef sites sampled for this study of the Kenyan microbiome. Source: Google Maps.

We investigated coral ecosphere within three of the five Kenya marine national parks i.e., Malindi (3°15′35.1′′S 40°08′40.0′′E), Mombasa (3°59′45.7′′S 39°44′50.1′′E) and Kisite-Mpunguti (4°42′54.0′′S 39°22′23.8′′E) (Figure 1). Located about 118 km north of Mombasa, Malindi Marine National Park and Reserve is the oldest MPA in Kenya having been gazetted in 1968 (McClanahan et al., 2010). This marine park is popular for glass bottom boat tours and snorkeling among other recreational touristic activities. It also experiences significant year-round discharge of freshwater and sediments with different types of nutrients from the Sabaki River which runs through a catchment area dominated by agricultural settlements (Katwijk et al., 1993; Munyao et al., 2003; Kitheka, 2019; Wanjeri et al., 2021). KWS wardens administering the MPA advised that there is considerable illegal fishing in the park at night spilling over from the “reserve,” an area adjoining the park where artisanal fishing is allowed. This represented the “exploited” site. The tidal range at these sites is approximately four meters. Mombasa Marine National Park was established in 1986 (Ngugi, 2002) with restrictions of protection being enforced commencing in 1991 (Tuda and Omar, 2012) and is the most visited of Kenya’s marine parks by both local and international tourists (Owens, 1978). It was considered the “polluted” site; owing to its proximity to the urbanized touristic city, the park experiences pollution from hotels, hospitals, domestic and industrial waste disposal (Mwangi et al., 2001; Okuku et al., 2011). Because Mombasa is the primary port serving inland eastern and central African countries, the park is also impacted by marine traffic including oil pollution and dredge-spoil dumping (Mwangi et al., 2001). On the southern coast, 90 km from Mombasa, the Kisite-Mpunguti site served as the “baseline” site. This protected area comprises of Kisite Marine National Park and Mpunguti Reserve created in 1973 and gazetted in 1978. It is bordered by sparsely populated coral islands and experiences the least human activities because it is 11 km offshore (Emerton and Tessema, 2001). There is no river runoff affecting the site (Okuku et al., 2019) and the most common activity here is snorkeling.

### Sampling

Cross-sectional sampling and *in situ* measurements were done in November and December of 2016 in all coral reef sites, 200 – 500 m from the shore at a depth of 1–2 m in low tides and within 15–20 cm of a healthy-looking *Acropora* spp. colony – the dominant species on the Kenyan coral reefs (McClanahan et al., 2007). A total of six samples were collected, three near-coral seawater, and three sediments. From each site, approximately 5L of seawater, and 10 cm column of sediments collected at the base of the same coral colony with a 10 mL syringe barrel. To assess inter-site variations at the time of sampling physicochemical parameters (water temperature, pH, salinity, and dissolved oxygen) were determined *in situ*, using portable multiprobe water quality meters as per the manufacturer’s instructions (YSI Inc., Yellow Springs, OH, United States). Finally, seawater samples were collected in triplicate 50-mL, for nutrient analysis. Samples were transported on ice to the laboratory for processing, typically within 3 h of sampling.

### DNA Isolation

From each site, 4 L of seawater was vacuum-filtered (VWR, West Chester, PA, United States) through a 0.2-μm pore size membrane (Pall Corporation, Port Washington, WI, United States) to capture microbial cells which were then added to a bead tube with lysis buffer. Microbial DNA from seawater samples was isolated with PowerWater^®^, and from sediment samples with PowerSoil^®^ DNA isolation kits according to the manufacturer’s instructions (Mo Bio, Inc., Carlsbad, CA, United States). Quality and quantity of DNA was checked by NanoDrop spectrophotometry. Suitability for sequencing of DNA samples for metagenomics analysis was confirmed with 1% agarose gel electrophoresis following 16S rRNA PCR amplification (Rohwer et al., 2002).

### Library Preparation and DNA Sequencing

Extracted DNA was subjected to whole-genome amplification by multiple displacement amplification (MDA) using the REPLI-g mini Kit according to the manufacturer’s instructions (QiaGen, Hilden, Germany), except that incubations were held for 10 h instead of the maximum recommended 16 h. Sequencing libraries were prepared from 1 μg of DNA according to the manufacturers’ preparation guide # 15036187 using the TruSeq DNA PCR-free library preparation kit (20015962/3, Illumina Inc.). Briefly, the DNA was fragmented using a Covaris E220 system, aiming at 350 bp fragments. Resulting DNA fragments were end-repaired, and the 3′ end adenylated to generate an overhang. Adapter sequences were ligated to the fragments via the A-overhang and the generated sequencing library was purified using AMPure XP beads (Beckman Coulter). The quality of the library was evaluated using the Fragment Analyzer system and a DNF-910 kit. The adapter-ligated fragments were quantified by qPCR using the Library quantification kit for Illumina (KAPA Biosystems/Roche) on a CFX384Touch instrument (Bio-Rad) before cluster generation and sequencing. A 200 pM solution of the individual sequencing libraries was subjected to cluster generation and paired-end sequencing with 150 bp read length using an S2 flowcell on the NovaSeq system (Illumina Inc.) using the v1 chemistry according to the manufacturer’s protocols. Base-calling was done on the instrument by RTA 3.3.3 and the resulting.bcl files were demultiplexed and converted to FASTQ format with tools provided by Illumina Inc., allowing for one mismatch in the index sequence. Sequencing was performed by the NGI SNP&SEQ Technology Platform in Uppsala, Sweden^1^.

### Bioinformatics Analyses

The raw Illumina reads were trimmed at Q5 threshold (MacManes, 2014), and the adapters were removed using fastp v0.19.5 (Chen et al., 2018). Reads were uploaded into the MGX metagenomics analysis platform v20200508 (Jaenicke et al., 2018) where paired-end reads were merged and quality-filtered at Q35 for subsequent analysis. Trimmed sequences were deposited to the European Nucleotide Archive under the project accession # PRJEB30838.

Taxonomic profiling was performed by applying the Lowest Common Ancestor (LCA) pipeline based on the Kraken tool, against the NCBI non-redundant (“nr”) database, enhanced by DIAMOND. Functional annotation was done using the clusters of orthologous groups (COG) of proteins database (Tatusov et al., 2000, 2001) with a BLASTX search of reads vs. the COG database applying MGX pipeline defaults (*E*-value cutoff 1e^–5^).

### Statistical Analyses

Output files from the MGX metagenomics analysis platform were exported to the R statistical environment (R Core Team, 2020) for analysis and visualization. Taxa and metadata files were merged using *phyloseq* version 1.28.0 (McMurdie and Holmes, 2013) and used in subsequent microbial community analyses. Rarefaction curves were estimated with the *ranacapa* package (Kandlikar et al., 2018) and plotted using *ggplot2* (Wickham, 2016). Estimates of α-diversity were measured within sample categories using *estimate_richness()* function of the *phyloseq* package. Non-metric multidimensional scaling (nMDS) and principal coordinates analysis (PCoA) ordinations of Bray-Curtis dissimilarity were performed using taxonomy relative abundance matrix by the package *vegan* 2.5–6 (Oksanen et al., 2019).

Abundance of each COG was counted as the sum of reads mapping to it (Tatusov et al., 2000), which was then normalized by the size of the dataset. COGs were assigned into functional categories. Functional diversity was estimated by the Shannon index based on COG richness and evenness (Johnson and Pomati, 2020). Bray-Curtis distance based on the relative abundances was calculated to represent functional composition variation among the samples (Bray and Curtis, 1957), and PCoA was used to visualize the relative differences. Normality of distribution of the relative abundances were assessed using the Shapiro-Wilk normality test. Differences in functional relative abundances were tested for statistical significance using Kruskal–Wallis test for COG categories, and chi-squared test for COG groups of proteins (*p* value < 0.05 as significance cut-off). False Discovery Rate (FDR) was corrected for using the Benjamini-Hochberg method (Benjamini and Hochberg, 1995).

Comparison of beta diversity between groups (both taxonomic and functional) was assessed by permutational multivariate analysis of variance (PERMANOVA) (Anderson, 2001) using the *adonis* test based on Bray-Curtis distances with 999 permutations in the package *vegan*.

## Results

### Inter-Site Variations in Coral Reefs

Physicochemical parameters characteristics are presented in Table 1. Measurements were comparable across the sites except for temperature and salinity recorded in Malindi that seemed to vary with the other sites: temperature recordings across the study sites ranged between 28.2°C (Kisite-Mpunguti) and 29.8°C (Malindi), while salinity was estimated as 27.6 ppt at Malindi compared to 30.1 ppt in both Mombasa and Kisite-Mpunguti. Dissolved oxygen and pH varied only slightly between the study sites.

**TABLE 1:** Measurements of physicochemical characteristics of the three coral reefs within the marine national parks at the time of sampling for metagenomic sequencing.

Variable	Kisite	Mombasa	Malindi
Temperature (°C)	28.2	28.6	29.8
pH (unit)	8.0	8.1	8.0
Dissolved oxygen (ppm)	8.5	8.3	8.2
Salinity (ppt)	30.1	30.1	27.6

The nutrients assessed, except for nitrites, were highest at Malindi, the “exploited” site, and lowest at Kisite-Mpunguti, the “baseline” site (Table 2). Although observed, differences in nutrient concentrations between the study sites were not statistically significant (ANOVA).

**TABLE 2:** Nutrient levels measurements of the three coral reefs within the marine national parks at the time of sampling for microbiome sequencing.

Nutrient	Kisite	Mombasa	Malindi	*p*
**NITRATES (NO_3_^–^ -N)**				
(mg/L + SD)	0.002 + 0.003	0.046 + 0.029	0.138 + 0.096	0.068
**NITRITES (NO_2_^–^ -N)**				
(mg/L + SD)	0.063 + 0.019	0.059 + 0.028	0.076 + 0.005	0.552
**AMMONIUM (NH_4_^+^ -N)**				
(mg/L + SD)	0.035 + 0.006	0.040 + 0.006	0.040 + 0.07	0.572
**PHOSPHATES (PO_4_^3–^ -P)**				
(mg/L + SD)	0.014 + 0.013	0.025 + 0.008	0.041 + 0.002	0.345

*Between-sites comparisons tested by ANOVA.*

### Microbial Diversity

A total of 211,966,893 reads from 6 samples were processed, 43% of which were classified taxonomically and functionally. Taxonomic assignment showed that four superkingdoms were represented, the majority (95.8% of the assigned reads) being Bacteria, followed by Eukaryota (1.7%), Archaea (1.3%), and Viruses (1.2%). Eukaryotic taxa included both unicellular (e.g., protists) and multicellular species (including *Acropora* spp. and fish) whose gametes or fragmented cells may have been sampled – associated reads were excluded from analysis.

**FIGURE 2:**
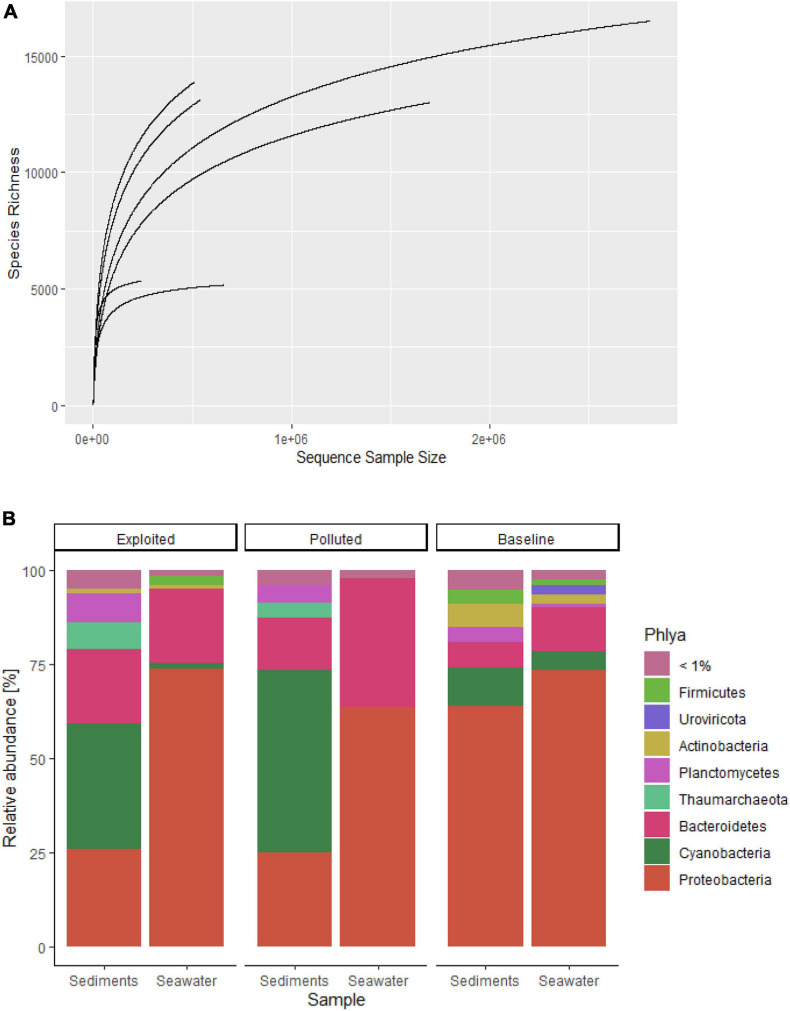
**(A)** Rarefaction curves for community species richness, and **(B)** stacked charts of the distribution of community phyla with relative abundance >1% sequenced from near-coral seawater and sediment samples from Kenya reefs at Malindi (Exploited), Mombasa (Polluted) and Kisite-Mpunguti (Baseline) Marine National Parks.

Rarefaction curves (Figure 2A) for most of the sequences from both datasets began to level off suggesting reasonable coverage of the microbial communities characterized with the processed reads. In all, 19,630 species (18,271 bacteria, 801 viruses and 558 archaea) belonging to 74 phyla were identified. Overall, Proteobacteria was the most dominant phylum with over 50% overall relative abundance (Figure 2B) followed by Bacteroides (20%) and Cyanobacteria (11%). Except for Kisite-Mpunguti metagenomes, Cyanobacteria was the most dominant phylum in the sediment sequences.

#### Bacteria

**FIGURE 3:**
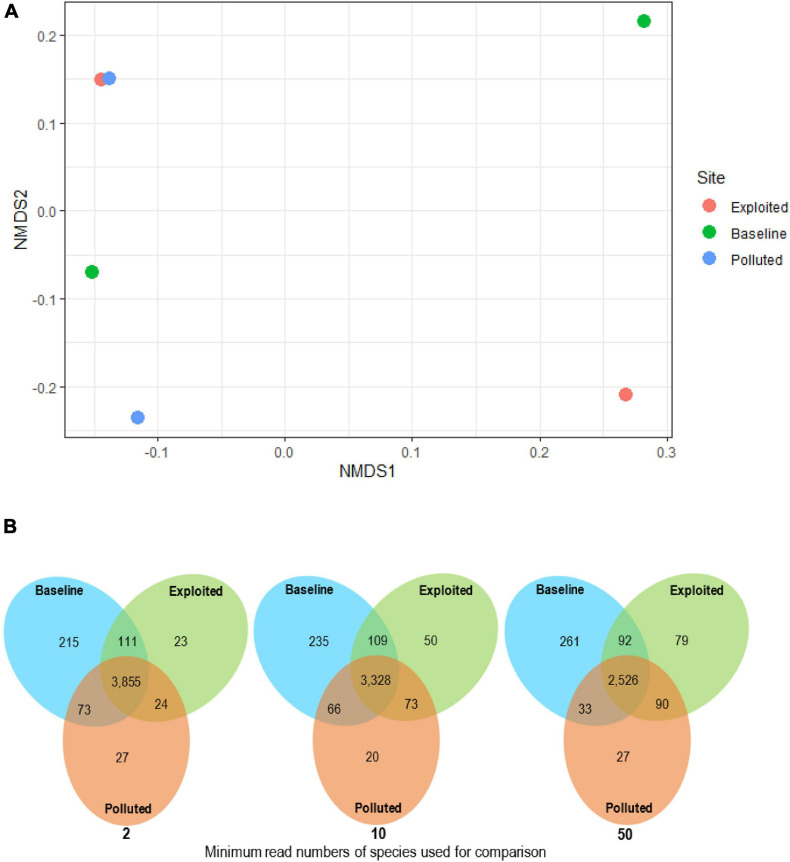
**(A)** Bacterial community composition analyzed with non-metric multidimensional scaling (nMDS) plots using Bray-Curtis dissimilarity for near-coral seawater and sediment samples from Malindi (peach circle; exploited), Mombasa (aqua circles; polluted) and Kisite-Mpunguti (green circle; baseline) national marine parks. For this nMDS plot, stress = 0.05, *r*^2^ = 0.998, and **(B)** venn diagrams showing the distribution of microbial species across study site metagenomes based on species with more than 2, 10, 50 reads.

A total of 18,271 bacterial species assigned to 53 phyla were classified from near-coral seawater and sediment samples. Classes belonging to Proteobacteria and Bacteroidetes phyla had the highest relative abundances. Among the Proteobacteria classes, Gammaproteobacteria, Betaproteobacteria and Deltaproteobacteria were the most dominant. Other classes that had a relative abundance higher than 1% belonged to the phyla Actinobacteria (Actinobacteria) and Firmicutes (Bacilli, Clostridia and Planctomycetes). Further inspection was made of the phyla Proteobacteria, Bacteroidetes and Actinobacteria, which are considered copiotrophic i.e., inhabiting nutrient-rich environments. In all, 13,903 copiotrophic species were assigned, the distribution of which did not cluster by site (Figure 3A) (PERMANOVA, *p* = 0.798). The “baseline” site had the highest number of unique species and the “polluted” site the lowest (Figure 3B).

**FIGURE 4:**
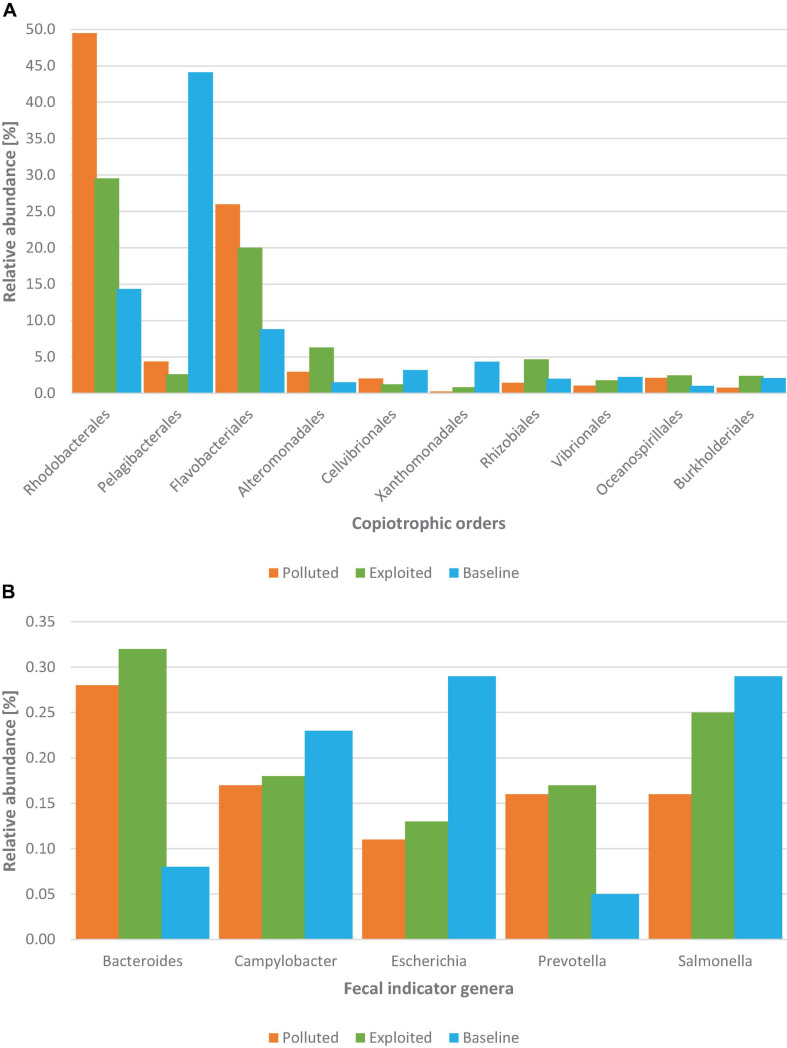
Distribution of **(A)** the top ten most abundant orders of the copiotrophic phyla (Proteobacteria and Bacteroidetes), and **(B)** distribution of common FIB genera from metagenomes of near-coral seawater and sediment samples from Kenyan reefs at the national marine parks classified as “polluted” (Bronze; Mombasa), “exploited” (Olive; Malindi) and “baseline” (Sky; Kisite-Mpunguti).

The first ten most abundant orders of the copiotrophic phyla assigned belonged to Proteobacteria and Bacteroidetes. Sequences assigned to Rhodobacterales (30.5%), Pelagibacterales (22.7%) orders of Proteobacteria, and the order Flavobacteriales (17.2%) of Bacteroidetes, were the most relatively abundant. By comparison, the relative abundance of each copiotrophic order was highest in either the “exploited” or “polluted” site except the orders Pelagibacterales, Cellvibrionales and Xanthomonadales whose relative abundances were highest in the “baseline” site (Figure 4A).

The relative abundances for sequences assigned to the common fecal indicator bacteria (FIB) genera (*Escherichia, Salmonella*, and *Campylobacter*) as well the alternative ones employed for microbial source tracking (i.e., *Bacteroides* and *Prevotella*) were less than 1% each (Figure 4B). The relative abundance of *Bacteroides* and *Prevotella* sequences from either “exploited” or “polluted” sites was more than three-fold higher than in the sequences from the “baseline” site. Relative abundances for *Campylobacter* and *Salmonella* sequences were comparable across the reef sites while that of *Escherichia* was more than two-fold higher in the sequences from the “baseline” site than in the sequences from either the “exploited” or “polluted” sites.

#### Archaea

**FIGURE 5:**
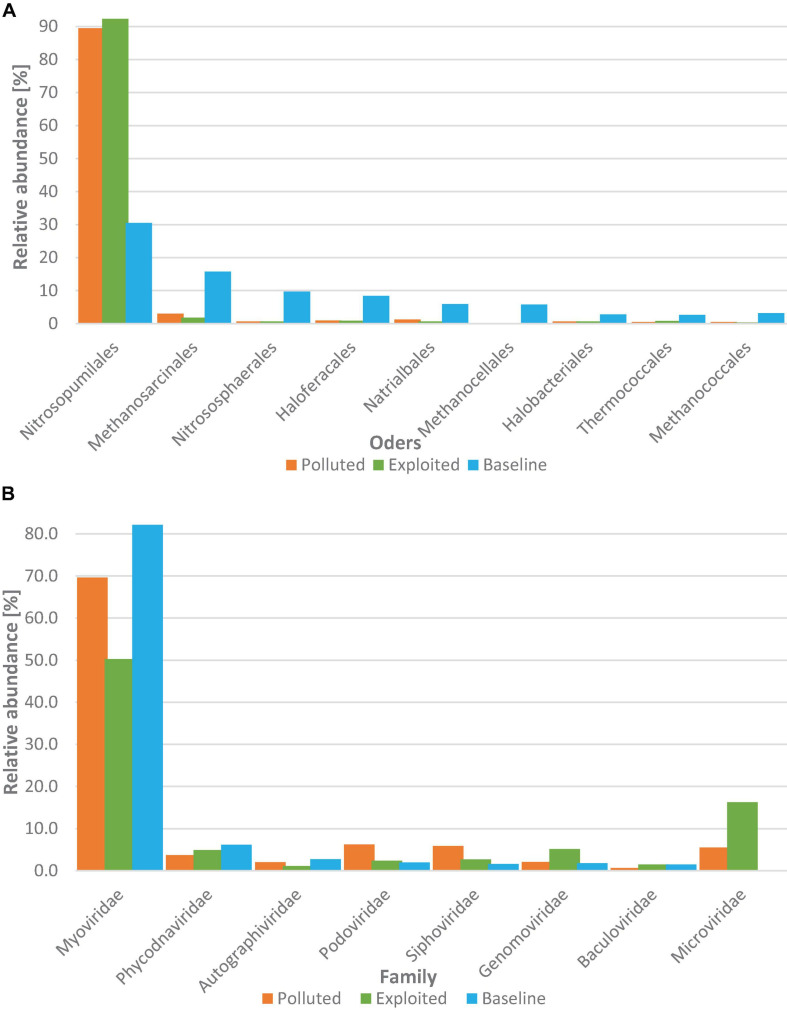
Distribution of **(A)** archaeal orders and **(B)** viral families with overall relative abundance >1% grouped by study site i.e., “polluted” (bronze), “exploited” (olive) and “baseline” (sky).

For the Archaea superkingdom, 558 species were assigned spread in eight phyla. Thaumarchaeota (88.5%) and Euryarchaeota (10.9%) were the most dominant phyla with the other six phyla accounting for only 0.7% of the sequences assigned. The distribution of archaeal orders with relative abundances above 1% is presented in Figure 5A. The order Nitrosopumilales recorded the highest relative abundance (>79%) followed by Methanosarcinales (5.1%). Sequences from the “baseline” site showed higher relative abundance for all orders except Nitrosopumilales (31%) for which the “exploited” and “polluted” site sequences were three-fold higher. Non-metric multidimensional scaling did not show any site-specific structuring of the Archaea superkingdom (PERMANOVA, *p* = 0.733).

#### Viruses

A 801 viral species, harbored in 11 phyla, were assigned to sequences from the near-coral seawater and sediment samples. Uroviricota (88.8%) dominated the sequences assigned. Only three other phyla i.e., Nucleocytoviricota (6.5%), Cressdnaviricota (2.5%), and Phixviricota (1.2%) had relative abundances of >1%.

Coliphages (viruses that infect coliform bacteria) constituted 85% of the sequences assigned at the family level. Myoviridae was the most dominant family (79.8%) (Figure 5B) and had higher relative abundance in the “baseline” (82.2%) than in the “exploited” (50.3%) and the “polluted” (69.7%) site metagenomes. The other coliphage families, Podoviridae, Siphoviridae and Microviridae were generally higher in the “polluted” (6.2, 5.9, and 5.5%) and “exploited” (2.4, 2.7, and 16.3%), than in the “baseline” (2.0, 1.6, and < 0.1%) site sequences. Up to 96% of the relative abundance at the species level comprised of cyanophages particularly viruses for *Prochlorococcus* and *Synechococcus* whose relative abundances were consistently higher (up to five-fold higher) in the “baseline” site sequences than in either the “exploited” or “polluted” site sequences.

### Microbiome Function

**FIGURE 6:**
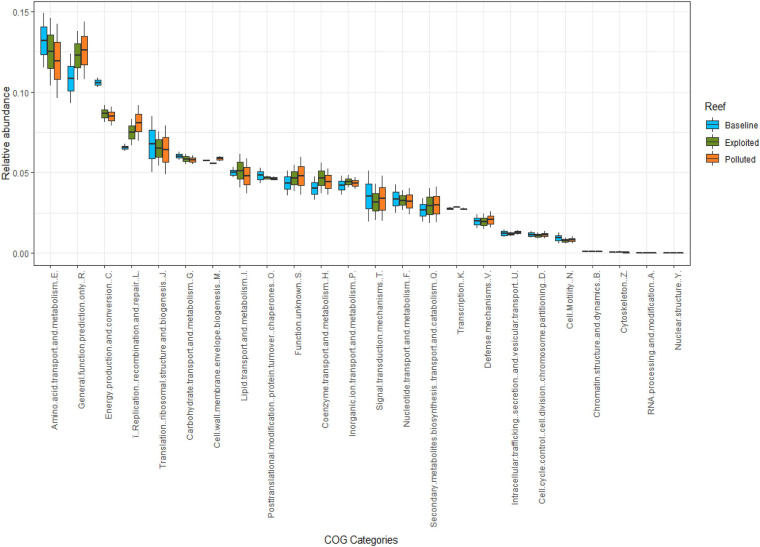
Relative abundances of COG functional categories compared between study sites i.e., “baseline” (sky blue), “exploited” (olive), and “polluted” (bronze).

A total of 4,584 clusters of orthologous groups of proteins (COGs) belonging to 24 COG categories were classified. Overall, the categories of amino acid metabolism and transport (E), general functional prediction only (R), energy production and conversion (C), and translation (J) had the most relative abundance together constituting 41% of all sequences. In contrast, categories of nuclear structure (Y, <0.01%), RNA processing and modification (A, 0.02%), cytoskeleton (Z, 0.05%), and chromatin structure and dynamics (B, 0.08%) had the least relative abundances. Between sequences from the reef sites (Figure 6), no statistically significant differences in relative abundances were observed (Kruskal-Wallis, *p* = 0.997), except for subtle variations in a number of COG categories including amino acid transport and metabolism (E), energy production and conversion (C), translation (J), carbohydrate transport and metabolism (G), and signal transduction (T) where the “baseline” site sequences seemed to have higher relative abundance compared to the “exploited” and “polluted” site sequences. In contrast, the relative abundances for the categories of general functional prediction only (R), replication and repair (L), cell wall membrane biogenesis (M), lipid metabolism (I), coenzyme metabolism (H), and secondary structure (Q) where higher in either “exploited” or “polluted” site sequences than in the “baseline” site sequences.

**FIGURE 7:**
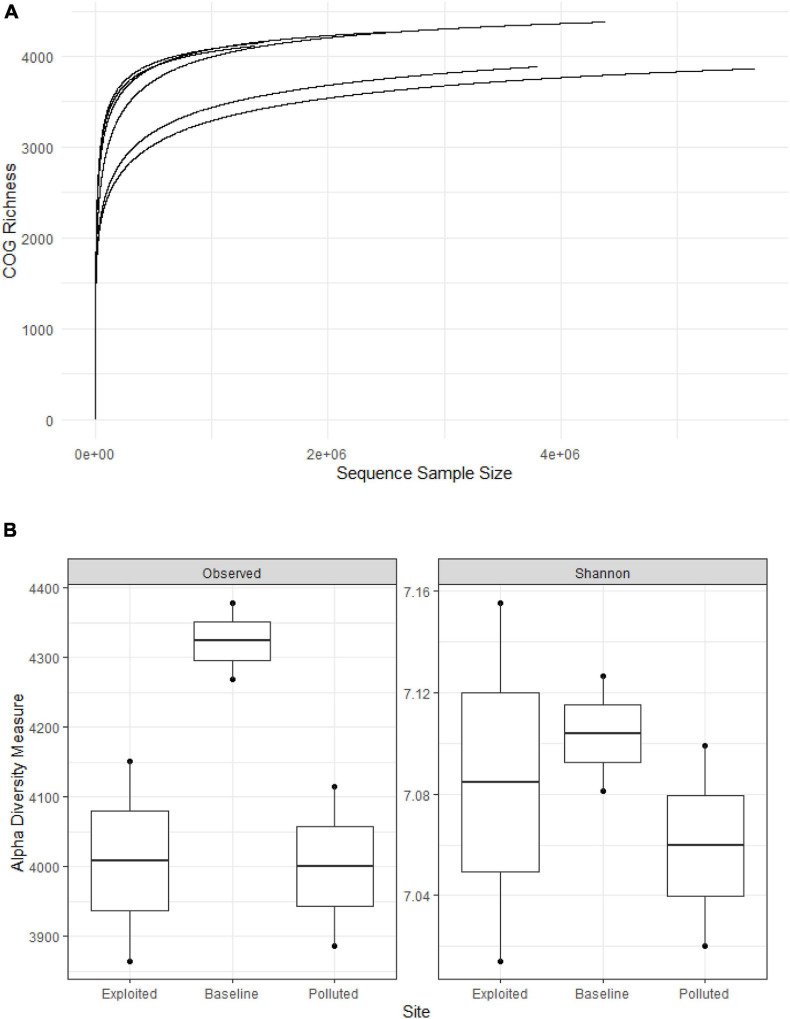
**(A)** Rarefaction curves and **(B)** plots of alpha diversity metrics for clusters of orthologous groups of proteins (COG) richness on the near-coral seawater and sediment metagenomes from Kenya reefs on the WIO.

The rarefaction curves (Figure 7A) shows the number of prevalent COGs identified with increasing sample number. The extent of sequencing showed that most COGs were recovered. The average per sample COG richness was estimated to be 4,111 + 205. The most prevalent COGs in all metagenomes corresponded to signal transduction histidine kinase (COG0642, 0.80%), dehydrogenases with different specificities (COG1028, 0.75%), NAD-dependent aldehyde dehydrogenases (COG1012, 0.68%), and RTX toxins and related Ca2 + -binding proteins (COG2931, 0.61%). The “baseline” site sequences recorded 4,324 observed COGs compared to 4,000 (“exploited”) and 4,008 (“polluted”), with a Shannon index of 7.12, compared to 7.06 (“exploited”) and 7.09 (“polluted”) (Figure 7B).

**FIGURE 8:**
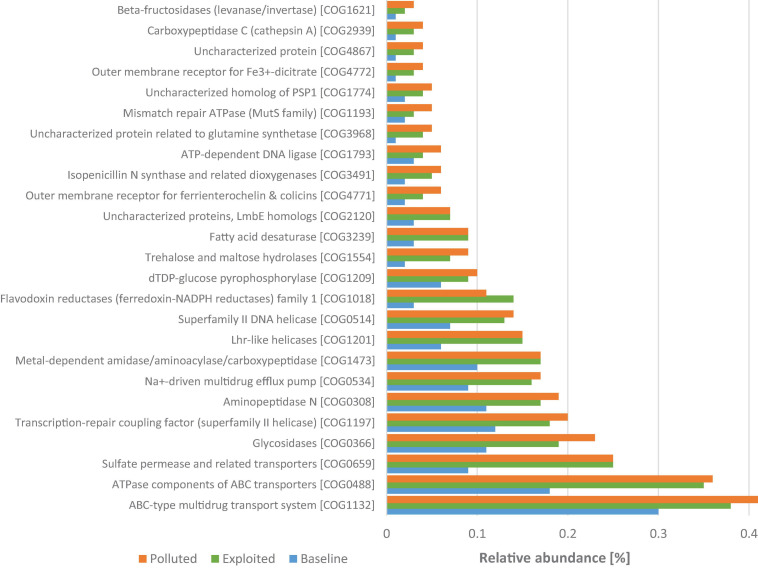
Comparison between Kenya coral reef sites of relative abundances of the 25 most represented COGs that were significantly enriched in the “exploited” and “polluted” sites.

The “baseline” sequences had 2,153 COGs that were significantly enriched, the most abundant of which were functions for energy production and conversion including, and transcription regulation COG2414, COG0674, COG1013, COG1145, COG1148, and COG3808. In contrast, 565 COGs were significantly enriched in the “exploited” and “polluted” metagenomes. In these sites, the most abundant COGs were dominated by predicted proteins related to defense mechanisms and removal or sequestration of unwanted compounds (e.g., COG1132, COG0534, COG3491, and COG0488), replication, recombination and repair (e.g., COG0514, COG1193, COG1793, and COG1201), and inorganic ion transport and metabolism (e.g., COG0659, COG4771, and COG4772) (Figure 8).

## Discussion

Microorganisms play a critical role to reef ecosystem health and resilience through mediating nutrient cycling, interactions with macro-organisms and provision of chemical cues influence the recruitment of diverse reef taxa. These processes may be impacted by environmental changes that cause microbial compositional and functional alterations which may, in turn, have consequences for the functioning of coral ecosystems (Glasl et al., 2018). Therefore, assessing changes in reef microbial communities and functional potential may provide early indicator of ecosystem impacts, and can help with development of diagnostic tools for monitoring shifts in coral reef health under different environmental states (Kisand et al., 2012; Bourlat et al., 2013; Glasl et al., 2017; Goodwin et al., 2017). We annotated near-coral seawater and sediment metagenomes from Kenyan coral reefs and compared microbial taxonomic and functional diversity across a gradient of the human activities the reefs are exposed to. Physicochemical parameters were also estimated to assess inter-site variations at the time of sampling.

Environmental variables at the coral reefs sampled fell mostly within the ranges considered suitable for coral growth in tropical oceans (Kannapiran et al., 2008; Henkel, 2010; Guan et al., 2015; Roik et al., 2016), except for salinity which was low (i.e., 27.6 – 30.1 ppt) compared to previous estimates (32 – 33 ppt) (Obura, 2001). This may have been due to high rainfalls during sampling and, especially for Malindi which had the lowest salinity occasioned by River Sabaki’s discharge into the ocean (Kitheka, 2019). During heavy rainfalls, salinity has been known to decrease down to 12 ppt in shallow reef flats (Obura, 2001). Except for nitrites, concentrations for all other nutrients assessed were higher at the “exploited” and “polluted” sites than at the “baseline” site suggesting influence by the surrounding human activities. These differences were, however, not statistically significant. Although temporal variations were not investigated in this study, current estimates in the “no-take” marine protected areas are typical of previous measurements taken about two decades ago (McClanahan, 1988; Obura, 2001). Being a cross-sectional survey, the reported measurements represented the quality of the coral reef water at the time of sampling, and not necessarily the stable state. A recent study modeling the contribution of natural and human factors in predicting water quality identified human influences as the random components of variation associated with site-based differences (Houk et al., 2020). The estimates in this study suggest, therefore, that at the time of sampling human impacts to the coral reefs in the “no-take” marine protected areas were undetectable or indistinguishable by water quality measurements.

Taxonomic composition, especially of the most abundant taxa, was largely consistent with other marine microbiota (Williamson et al., 2012; Sunagawa et al., 2015; Godoy-Vitorino et al., 2017; Santoro et al., 2019). The estimate for species richness was within the same order of magnitude as the previous metagenomic estimate for global oceans (Sunagawa et al., 2015). Although the majority of assignments were shared, there were sequences unique to each site whereby Kisite-Mpunguti (“baseline”) had the highest number, reinforcing the hypothesis that rather than every microbe being ubiquitous, taxa are selected or structured by the environment (Flaviani et al., 2018). The seawater and sediment samples collected within coral reefs generally harbored microbial composition consistent with the typical taxa enriched by metabolic products of corals i.e., copiotrophic bacterial groups (including Gammaproteobacteria, Betaproteobacteria, and Bacteroidetes), ammonia oxidizing archaea (AOA) of the order Nitrosopumilales, and phages for the copiotrophic bacteria (coliphages) i.e., Podoviridae, Siphoviridae, and Microviridae (Sunagawa et al., 2010; Kelly et al., 2014; Godoy-Vitorino et al., 2017; Thurber et al., 2017; Weber et al., 2019). Although coral reef seawaters are typically dominated by copiotrophic bacterial community due to increased organic matter from mucus and other nutrients released by corals (Bourne and Webster, 2013), between-sites differences observed in the bacterial relative abundances seemed to correspond to the varying magnitudes of human activities around the coral reef sites compared. Most copiotrophic bacteria, especially the most abundant ones, had higher relative abundances in either the “exploited” or “polluted” than in the “baseline” site sequences. This is significant considering that copiotrophic bacteria thrive in high nutrient environments and include taxa utilized in water microbiology as fecal indicators (Buccheri et al., 2019). Also, the genera *Bacteroides* and *Prevotella* exhibited higher relative abundance in both the “exploited” and “polluted” than in the “baseline” site sequences – these genera are increasingly exploited as alternative groups to replace the less reliable traditional FIB. Contrary to expectations, the relative abundance for members of the order Pelagibacterales and the traditional FIB genera *Escherichia, Salmonella*, and *Campylobacter* were higher in the “baseline” than in the “exploited” and “polluted” site sequences (Figure 4). Pelagibacterales belongs to the class Alphaproteobacteria whose ubiquitous members, although heterotrophs, are known to thrive at low nutrient concentrations typical of open ocean conditions (Kelly et al., 2014; Zhao et al., 2017). The higher relative abundance of this group in the “baseline” than in the “exploited” and “polluted” site sequences may have been, therefore, indicative of differences in nutrient levels at the sites perhaps due to the surrounding human activities. As for the FIB genera, it is not clear why relative abundances did not correlate with human impact gradients at the coral reef sites. One possible explanation is what has been termed as “connectivity footprint” – the phenomenon that even MPAs thousands of kilometers away can be influenced by human activities due to dispersal by ocean currents (Robinson et al., 2017). The “baseline” site, which recorded highest relative abundances for the traditional FIB genera was sampled in December during the north-east monsoon (NEM) which is characterized by the convergence of the southward flowing Somali current (SC) with the northward flowing East African coastal current (EACC) and upwelling along the Kenyan coast (Heip et al., 1995; Mayorga-Adame et al., 2016). It is conceivable that the fecal bacteria originated elsewhere and transported to the “baseline” site by the SC as the site is south to the “exploited” and “polluted” sites. However, if this was the case, similar relative abundances would have been observed of the other taxa, for instance copiotrophic bacteria and coliphages. An alternative explanation is that FIB detected at the “baseline” site were of sources not related to human fecal contamination. FIBs are shed in the feces of many different animals (Harwood et al., 2014), and naturalized or environmentally adapted strains of FIB can persist and multiply in a broad range of habitats far removed from their natural reservoir of the animal gut (Devane et al., 2020). It is for these reasons that traditional FIB genera are generally considered unreliable as fecal indicators.

Coliphages i.e., viruses that infect coliform bacteria, are considered better predictor of fecal pollution in seawater than FIBs (Burbano-Rosero et al., 2011) because they outlive their bacterial hosts, they can thrive in marine environments unlike some of their anaerobic hosts, they are more resistant to disinfection and diffuse further distances from pollution sources (Ebdon et al., 2012; Bari and Yeasmin, 2014). In this study, relative abundances of all coliphage families assessed, except one, were higher in the “exploited” and “polluted” site sequences than in the “baseline” site sequences. The coliphage family, Myoviridae, had higher relative abundance in the “baseline” site sequences than in the “exploited” and “polluted” site sequences. The cause for this exception is not clear but it may have been influenced by the relative abundance of *Escherichia* which was higher in the “baseline” site sequences and which members of the Myoviridae family are known to preferentially infect (Tao and Rao, 2019). Cyanophages too recorded relative abundances commensurate to the bacteria they infect, cyanobacteria. Phages for *Prochlorococcus* and *Synechococcus* were up to five-fold higher in the “baseline” site sequences than in either the “exploited” or “polluted” site sequences. *Synechococcus* and *Prochlorococcus* are considered the most important primary producers in the tropical oceans, responsible for a large percentage of the photosynthetic production of oxygen (Waterbury et al., 1979; Biller et al., 2015; Kim et al., 2018) and it is thought that their phages mediate their population sizes and evolutionary trajectories (Sullivan et al., 2005). Being autotrophs, members of these genera are usually found in great abundance in ocean zones low in nutrients (Dinsdale et al., 2008; Kelly et al., 2014). These findings call for further investigations to establish the stability of these trends over time and space for the potential utilization of these virus groups, especially the coliphages, as surrogates for human impacts in coral reefs.

Among the Archaea groups, the AOA order Nitrosopumilales had the most obvious correlation with human activity gradient; in comparison to the “baseline” site sequences, the order was three times higher in the “exploited” and “polluted” site sequences. Members of this order are key players in nitrification processes (Alvarez-Yela et al., 2019) that have been shown to seek nutrient-rich environments where they utilize urea and ammonia as substrate (Bayer et al., 2016). All the other orders with over 1% relative abundance were higher in the “baseline” than in the “exploited” and “polluted” site sequences.

The COG richness observed in this study was slightly higher but comparable to the others previously reported by studies of global oceans and coral ecosystems (Carlos et al., 2016; Varasteh et al., 2020; Zhang et al., 2020). The predicted proteins for signal transduction histidine kinase which enables microbes to sense, respond, and adapt to a wide range of environments, stressors, and growth conditions (Skerker et al., 2005) were the most abundant. Furthermore, the coral ecosphere sequences had overrepresentation of predicted proteins related to replication, recombination and repair especially transposases that play a role in adaptation to diverse environments (Vigil-Stenman et al., 2017), and predicted proteins for energy production and conversion which is an adaptation for high nutrient uptake and synthesis needed in low nutrient environments (Gudhka et al., 2015). These are likely strategies for optimizing rapid growth in the presence of labile nutrients – an adaptation of copiotrophs e.g., Gammaproteobacteria in environments of high levels of stress factors and nutrients (Kisand et al., 2012). These observations suggest that the three coral reefs had substantial levels of stressors and nutrients. Nonetheless, there were subtle differences across the human impacts’ gradient suggesting heterogeneity, possibly anthropogenically influenced.

Kisite-Mpunguti marine park (“baseline”) sequences were enriched, more than the “exploited” and “polluted” sequences, with protein groups essential for functions related to uptake and synthesis of nutrients and transcription – a common observation among oligotrophic organisms as an adaptation to low levels of nutrients (Lauro et al., 2009).

Conversely, metagenomes from Malindi (“exploited”) and Mombasa (“polluted”) marine parks had overrepresentation of COGs suggestive of mitigation of environment stressors. Here the enriched COGs included, for instance, proteins related to defense mechanisms such as transporters that prevent intracellular accumulation of toxic compounds (Wilkens, 2015), which serves as the major defense mechanism against antimicrobial compounds (Lubelski et al., 2007). For instance, a recent study assessing antibiotic resistance genes along a pollution gradient also found the highest abundance of transporters at the most polluted site (Chen et al., 2019). COGs for DNA replication, recombination and repair were also significantly overrepresented suggesting exposure to agents of DNA damage (Buckley et al., 2020; Zhang et al., 2020). Together, these observations reflect hostile environmental conditions. It is potentially significant that the genes that ward off environmental insults are significantly enriched in the metagenomes from Malindi and Mombasa marine parks, the sites with known degradative human activities ashore (Katwijk et al., 1993; Heip et al., 1995; Okuku et al., 2011; Ongore et al., 2013; Wanjeri et al., 2021), and not in Kisite-Mpunguti marine park which is situated farther from human settlements thereby potentially experiencing limited direct human impacts. Similar observations have been made, in a study comparing microbial functions of a highly polluted and protected marine environments (Kisand et al., 2012), and attributed to adaptations to an environment characterized by highly heterogeneous, variable and unfamiliar stimuli from anthropogenic source. These observations provide insights into the functional shifts contributed by human impacts, and potential COGs that may be utilized as indicators of marine health generally, and coral reef specifically. However, being DNA-based observations, these findings need validation by transcriptomics and proteomics approaches to confirm the actual proteins that are overexpressed in the respective sites.

## Conclusion

Our results reveal subtle taxonomic and functional between-sites heterogeneity suggestive of human influence. The environmental significance of inter-site variations observed in this study needs to be confirmed through robust studies controlling for seasonal variations and spatial stability. Nonetheless our findings underscore the potential of microbial ecology in informing strategies of marine monitoring. We therefore recommend integration of microbial sampling in the management of national marine parks and the respective services they provide by both the national and county government agencies.

## Data Availability Statement

The datasets presented in this study can be found in online repositories. Trimmed sequences are deposited to the European Nucleotide Archive under the study accession number PRJEB30838.

## Author Contributions

SW designed the study, conducted field sampling and laboratory processing, performed the bioinformatics analyses, and drafted and revised the manuscript. HG designed the study, coordinated sequencing, performed the bioinformatics analyses, and drafted the manuscript. EV, OK-L, NW, EB-R, AM, and SV helped design the study and edited the manuscript. All authors contributed to the article and approved the submitted version.

## Conflict of Interest

The authors declare that the research was conducted in the absence of any commercial or financial relationships that could be construed as a potential conflict of interest.

## Footnotes

1 www.sequencing.se
